# The Disappearing Murmur: Systolic Anterior Motion of the Mitral Valve Leaflet in a Non-hypertrophic Cardiomyopathy Patient

**DOI:** 10.7759/cureus.2855

**Published:** 2018-06-21

**Authors:** Syed Rafay Ali Sabzwari, James R Kimber, Hiwot Ayele, Nimra Khan, Tarick Sheikh, Ghulam Akbar, Bruce Feldman

**Affiliations:** 1 Cardiology Fellowship, Lehigh Valley Health Network, Allentown, USA; 2 Cardiology, Lehigh Valley Health Network, Allentown, USA; 3 Medicine, Florida Hospital, Orlando, USA; 4 Internal Medicine, Lehigh Valley Health Network, Allentown, USA

**Keywords:** systolic anterior motion, hypertrophic obstructive cardiomyopathy, mitral regurgitation, mitral regurgitation and heart failure

## Abstract

Systolic anterior motion (SAM) of the mitral valve is a well-known phenomenon associated with left ventricular outflow tract obstruction and hemodynamic compromise. This finding may occur in patients with or without hypertrophic cardiomyopathy. In this report, a patient with no prior medical history presented to the hospital with left-sided chest pain and high-risk echocardiogram (ECG) findings. Left heart catheterization with coronary angiography was negative for coronary artery disease. His initial examination was significant for a systolic murmur due to the underlying SAM, as demonstrated by transthoracic echocardiogram. During his hospitalization, he developed acute heart failure syndrome as a result of dynamic outflow tract obstruction. He was treated with fluid resuscitation with a resolution of his hemodynamic compromise. On a follow-up examination, there was no murmur and SAM was no longer present on echocardiogram. This case demonstrates the importance of recognizing the clinical manifestations of SAM as well as its role in maintaining an appropriate hemodynamic status.

## Introduction

Systolic anterior motion (SAM) of the mitral valve leaflet has been well-described among patients with hypertrophic cardiomyopathy (HCM); however, this phenomenon also occurs in a population of patients without HCM. According to a Japanese study, investigating the incidence of SAM using resting echocardiography, the prevalence of SAM among patients without HCM was 0.3% [1]. In the same study, the presence of SAM was associated with advanced age (mean age: 63 years), other echocardiographic features, including a sigmoid septum, and lower left ventricular outflow tract (LVOT) pressure gradients as compared to patients with known HCM [1]. Additionally, SAM is often associated with asystolic murmur on physical examination. While the presence of SAM is often due to the pathology of the mitral valve, chordal SAM has also been described in case series and reports. Imaging modalities, such as transesophageal echocardiography (TEE) and cine cardiac magnetic resonance imaging (MRI), are valuable in identifying anatomic variants of the mitral valve apparatus associated with this phenomenon.

## Case presentation

We present a case of a 34-year-old male with no past medical history who presented to the emergency department (ED) with several hours of left-sided chest pain and headaches. The symptoms were preceded by one week of viral prodrome with rhinorrhea, sore throat, mild fevers, and poor oral intake. He appeared acutely distressed due to chest pain, with a blood pressure of 73/43 mmHg, heart rate 116 bpm, respiratory rate 20/min, oxygen saturation 100% on two liters of supplemental oxygen via the nasal cannula. The cardiac examination was significant for a Grade III/VI pansystolic murmur, best heard at the apex with radiation to the axilla. The ECG revealed high-risk findings with ST elevation in aVR and reciprocal depressions in the remaining leads. Initial labs showed abnormalities of bicarbonate, creatinine, and lactic acid.

**Figure 1:**
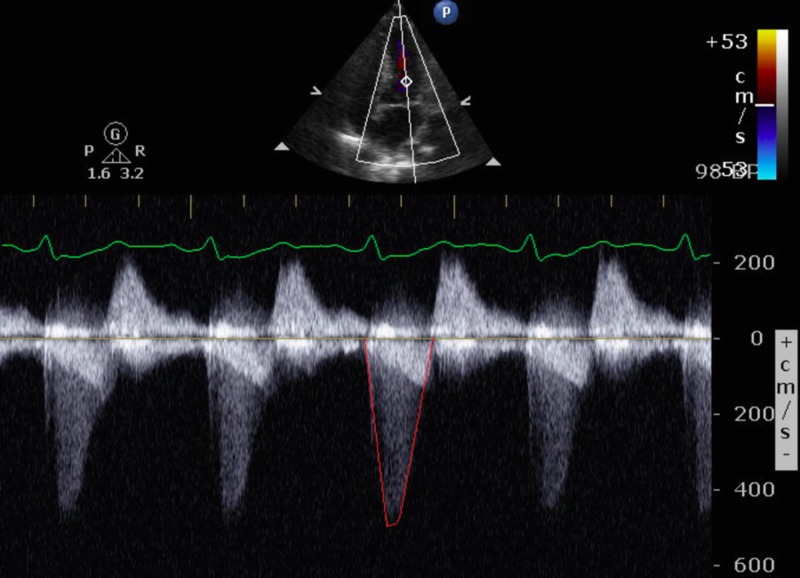
Transthoracic echocardiographic image showing mitral regurgitation signal (red outline) with continuous wave Doppler

**Figure 2:**
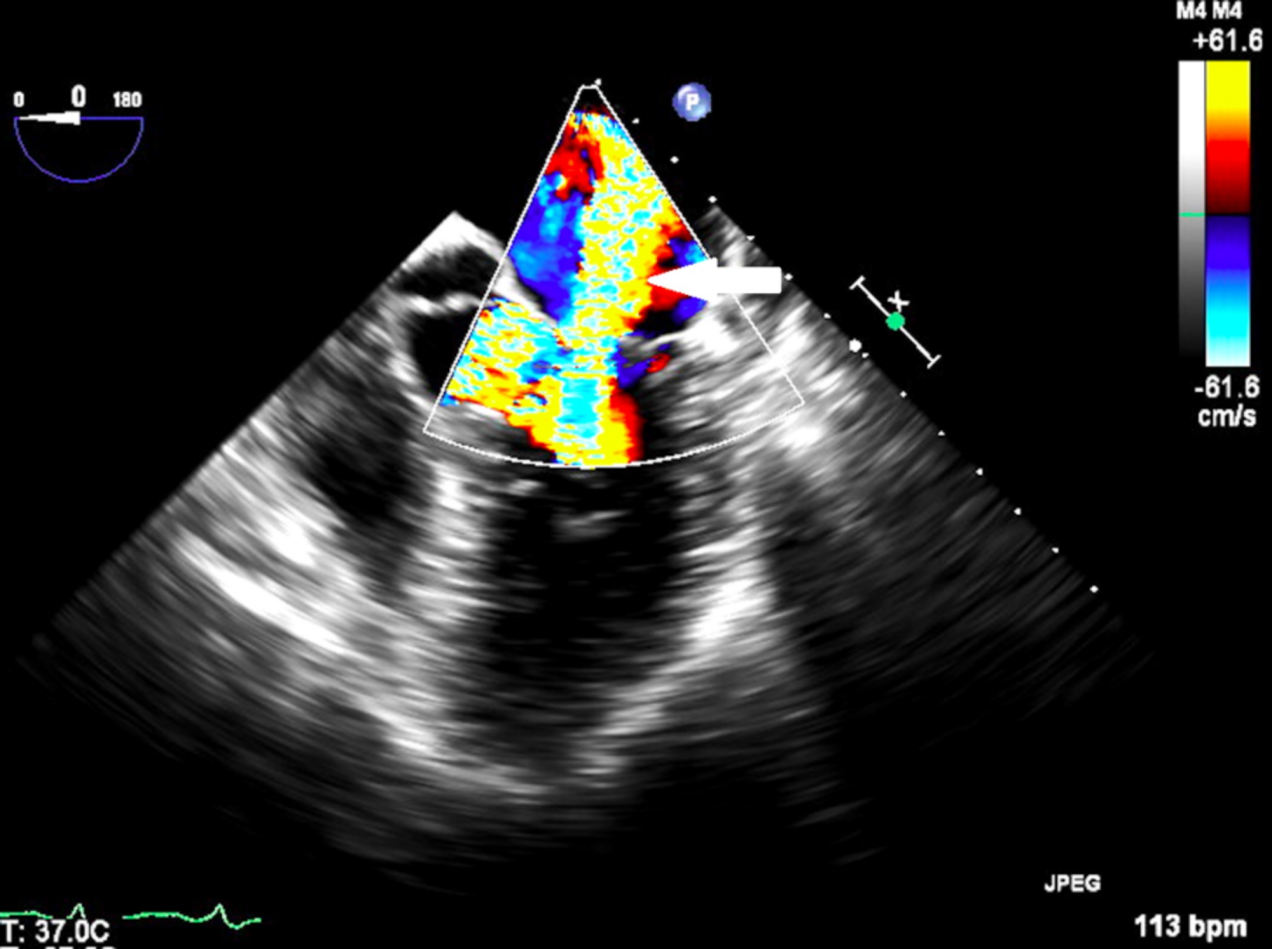
Transesophageal echocardiographic image showing mitral regurgitation with color Doppler (white arrow)

**Figure 3:**
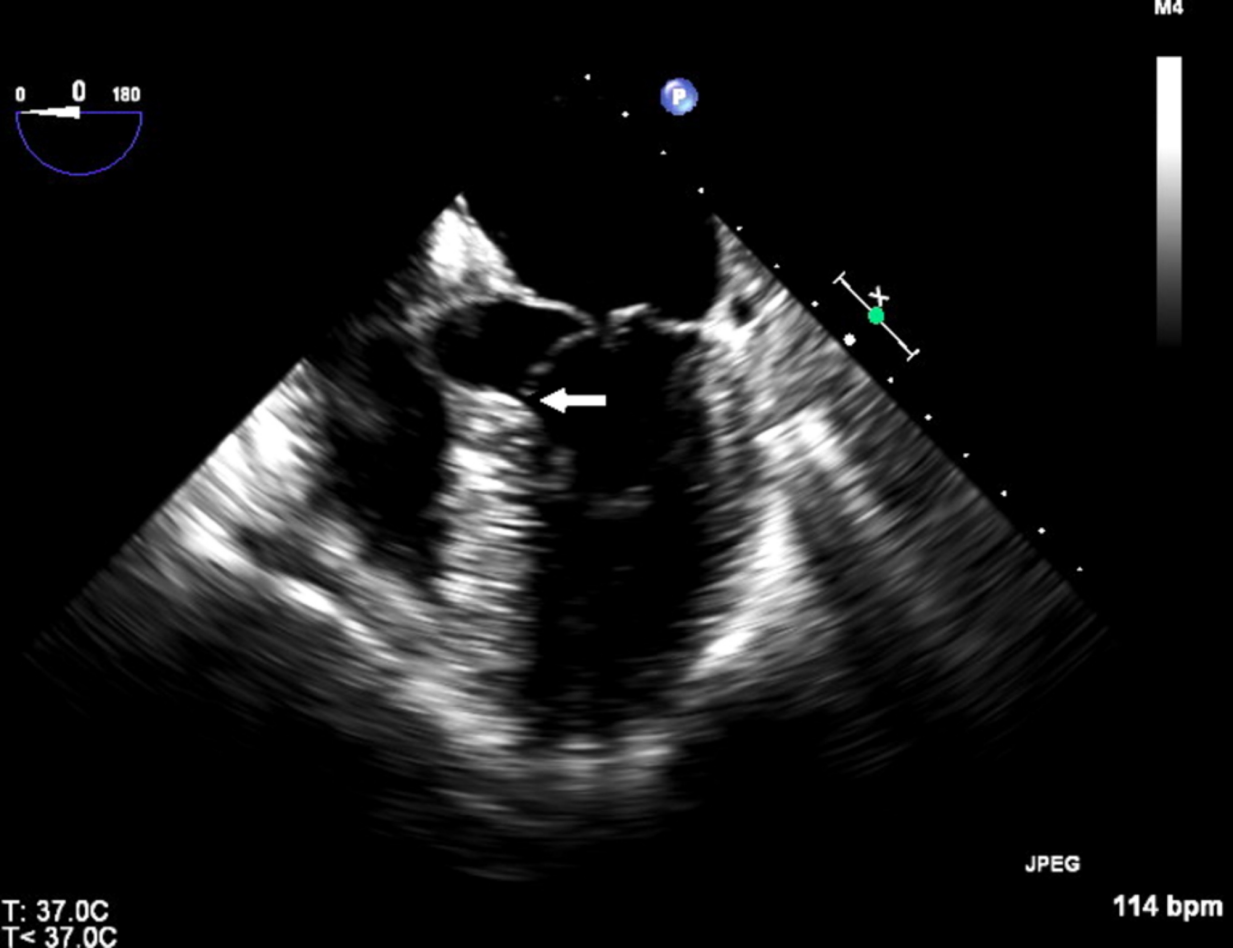
Transesophageal echocardiographic image showing systolic anterior motion of the anterior mitral valve leaflet (white arrow)

**Figure 4:**
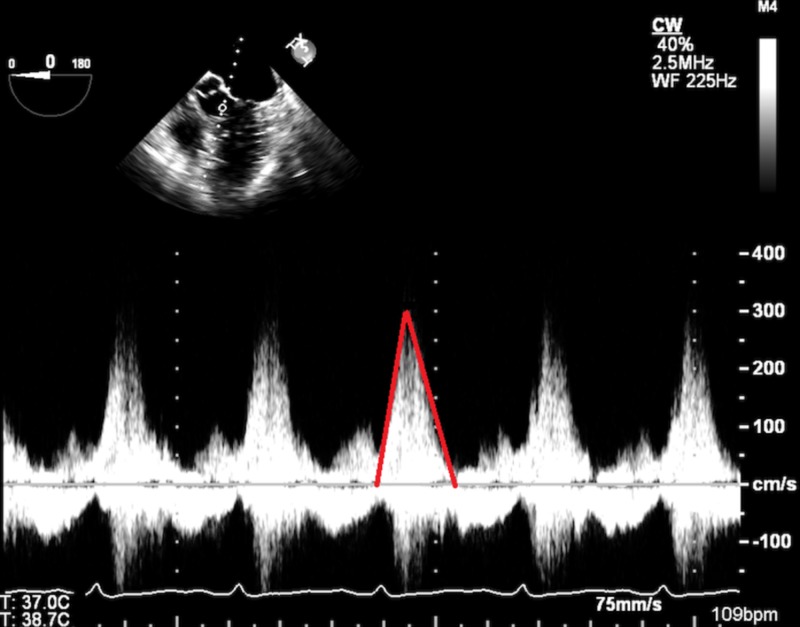
Transesophageal echocardiographic image showing increased left ventricular outflow tract velocity with continuous wave Doppler (red outline)

Due to this constellation of high-risk ECG, hemodynamic instability, and chest pain refractory to medical therapy, the patient was taken for emergent cardiac catheterization. A coronary angiogram and left ventriculography showed only minor luminal irregularities, hyperdynamic systolic function, 2+ mitral regurgitation, and a left ventricular end-diastolic pressure of 22 mmHg. On right heart catheterization, the right atrial pressure was 13 mmHg, the right ventricular pressure was 50/15 mmHg, the pulmonary artery pressure was 50/22 (mean 34) mmHg, and the pulmonary capillary wedge pressure was 29 (v wave 51) mmHg. A transthoracic echocardiogram showed moderate mitral regurgitation (MR) with thickened leaflets and an echodensity in the submitral apparatus suspicious for torn chordae tendineae versus ruptured papillary muscle (Figure 1). Subsequently, an urgent transesophageal echocardiogram (TEE) was performed and showed severe eccentric MR (Figure 2) associated with SAM (Figure 3) of a structurally normal mitral valve and no evidence of left ventricular or septal hypertrophy. The peak gradient was measured to be 50 mmHg across the left ventricular outflow tract (LVOT) (Figure 4).

**Figure 5:**
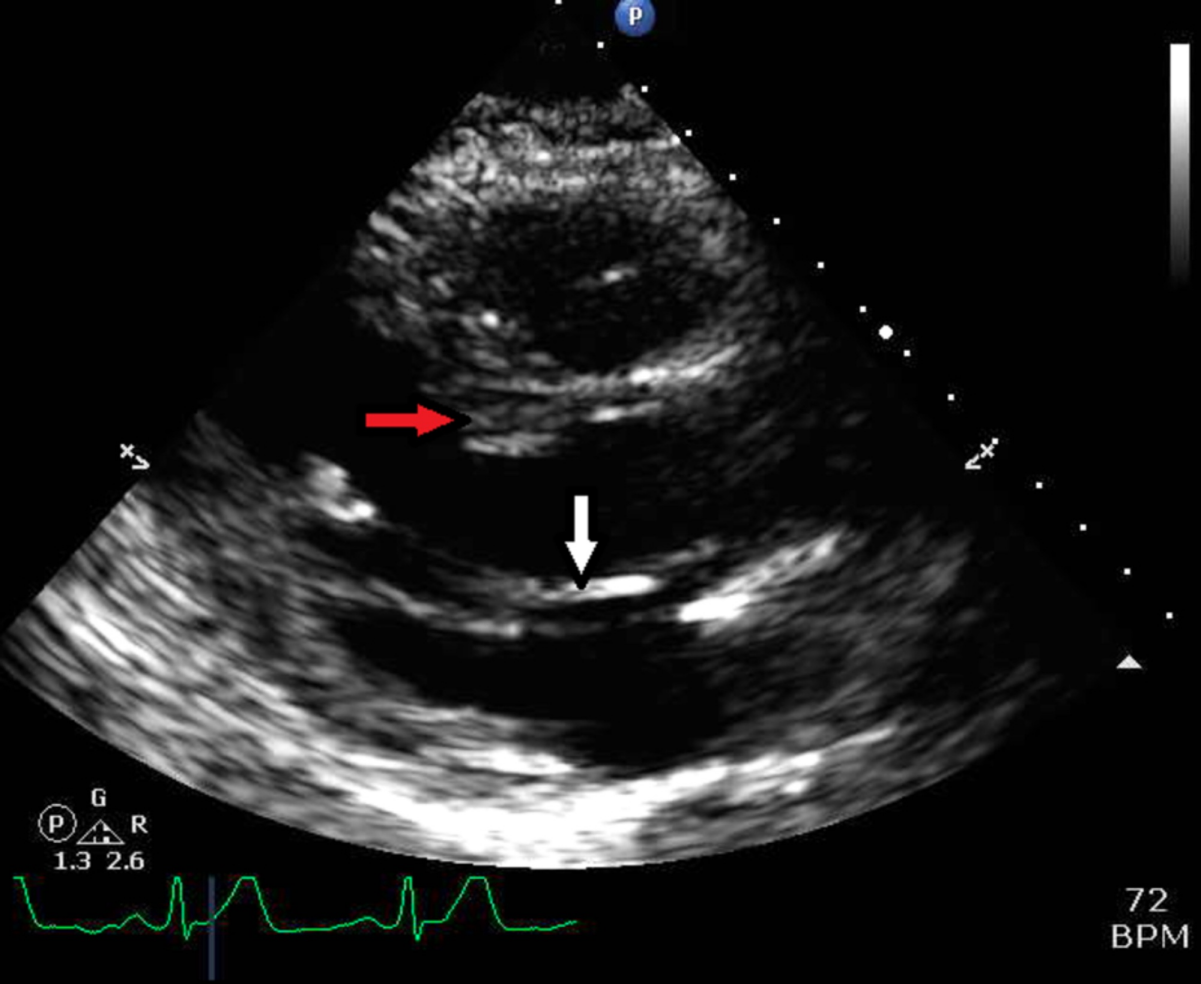
Next-day transthoracic echocardiographic image showing no contact between anterior mitral valve leaflet (white arrow) and the interventricular septum (red arrow)

**Figure 6:**
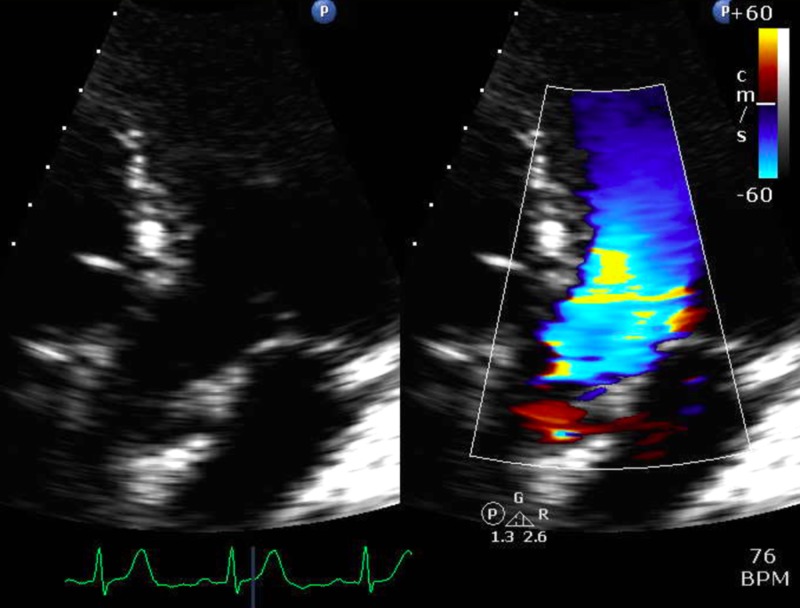
Next-day transthoracic echocardiographic image without left ventricular outflow tract obstruction

Following these studies, he required treatment with fluid resuscitation and phenylephrine infusion to support his blood pressure. Over the course of 12 hours, the patient received four liters of normal saline with an improvement in his hemodynamics. His subsequent physical examination was negative for a systolic murmur and showed no new cardiac findings. A limited TTE was repeated and showed no evidence of SAM (Figure 5) or MR (Figure 6), and no dynamic LVOT obstruction at rest. A cardiac MRI did not show delayed gadolinium enhancement to suggest myocarditis or a scar. The study did show a mild increase in the mid-inferior and basal anterior septal thickness to 15 mm without a resting LVOT obstruction.

On the second day of hospitalization, the patient was started on a low-dose beta blocker, which he tolerated well. The remainder of his stay was uncomplicated, as he remained asymptomatic with a resolution of the lab abnormalities.

## Discussion

This patient had an elongated anterior mitral valve leaflet, which led to an increased LVOT gradient and hemodynamic compromise in the setting of a viral syndrome with dehydration. He manifested symptoms of acute heart failure syndrome with severe MR and elevated wedge pressure as a result of his dynamic outflow tract obstruction. The occurrence of this phenomenon has been described in older patients, but it is unusual in a young patient without evidence of HCM and has not been previously reported in the literature.

Once identified, treatment consists of maintaining adequate preload through volume resuscitation. Patients also benefit from increased diastolic filling time from the negative chronotropic effect of beta blockers. Uematsu et al. showed that 75% of non-HCM patients with SAM had a sigmoid septum. As such, there may be a utility in therapy with atenolol and cibenzoline in reducing LVOT gradients [1-2]. In this case, volume resuscitation alone was sufficient to restore the patient’s normal resting hemodynamics and was able to resolve the clinical and echocardiographic findings of dynamic LVOT obstruction. In cases in which there is a significant hemodynamic compromise, volume resuscitation is the preferred therapy over vasopressors to avoid further worsening of gradients. This highlights the importance of urgent imaging and the identification of SAM in its contribution to LVOT obstruction in appropriate resuscitative management.

Patients who have SAM without HCM require routine follow-up echocardiography. This evaluation may demonstrate high or low LVOT pressure gradients. Both variants have the potential to develop a high-pressure gradient under various conditions (e.g., volume depletion, tachycardia). An additional follow-up examination should include exercise evaluation and auscultation maneuvers (Valsalva) to evaluate for clinical evidence of an LVOT obstruction.

It has been previously reported that the finding of chordal SAM during peak exercise and a small LV end-systolic diameter were significant predictors of LVOT obstruction [3-5]. Other studies suggest that the anterior position of papillary muscles and mitral leaflet elongation are responsible for SAM and LVOT obstruction [3-5]. Hence, 3D echocardiographic imaging and computed tomography have been useful for defining the anatomy of the mitral valve and its subvalvular apparatus responsible for SAM.

Maron et al. reported that the prevalence of SAM without asymmetrical septal hypertrophy or the transposition of the great vessels was 0.4% among 721 patients with a wide variety of cardiac diseases other than HCM [6]. Pearson et al. reported that the prevalence of chordal SAM without asymmetrical septal hypertrophy or congenital heart disease was 3.9% among 1552 patients who underwent an echocardiography for clinical reasons and 1% among 100 normal volunteers [7]. Zywica et al. reported that 16% of 280 patients who underwent exercise echocardiography presented with chordal SAM [8]. Several studies have reported the postoperative incidence of SAM as ranging from 2.3% to 14% [6-8].

## Conclusions

This case demonstrates the importance of recognizing the clinical manifestations of SAM and its role in maintaining an appropriate hemodynamic status. A hyperdynamic ventricle with reduced diastolic filling time and reduced preload predisposes to SAM. Prompt diagnosis is crucial to institute appropriate fluid resuscitative measures and to avoid vasopressors that would worsen LVOT gradients and lead to hemodynamic deterioration. Echocardiography and cardiac MRI are great modalities to diagnose this phenomenon and to follow up hemodynamics. Since there is no HCM, the risk of sudden cardiac death is not the same as in HCM patients and an implantable cardiac defibrillator is not indicated. However, appropriate instructions on maintaining good hydration and instituting negative chronotropic agents are necessary.
